# MixNet: A scale-adaptive method for multivariate time series forecasting

**DOI:** 10.1371/journal.pone.0349573

**Published:** 2026-05-26

**Authors:** Xinhan Wang, Bowen Zhao

**Affiliations:** 1 Southwest China Institute of Electronic Technology, Chengdu, China; 2 School of Computing and Artificial Intelligence, Southwest Jiaotong University, Chengdu, China; University of Queensland - Saint Lucia Campus: The University of Queensland, AUSTRALIA

## Abstract

Time series forecasting is a critical task with widespread applications in industrial domains and daily life, including weather prediction, long-term energy consumption planning, and marketing analysis. Nevertheless, effectively extracting salient temporal patterns and exploring dependencies within multivariate time series remains a challenge. This paper focuses on multivariate time series forecasting, a common and pivotal issue in numerous analytical tasks. To address the complexity and high variability inherent in multivariate time series, we propose a scale-adaptive multi-head attention mechanism based on a hybrid mixture of experts network. Building on this mechanism, we develop MixNet, a novel architecture designed to achieve flexible feature extraction across diverse types of time series data. Furthermore, to tackle the difficulty in capturing inter-variable dependencies, we introduce a dedicated multivariate time series embedding (MTSE) scheme integrated with learnable positional encoding. This approach aims to comprehensively model the dependencies among variables, thereby enhancing overall forecasting performance. Experimental results demonstrate that MixNet outperforms several state-of-the-art methods on seven benchmark datasets from primary domains.

## Introduction

Time series data is a fundamental component of data analysis and has a wide range of applications in industry and daily life. As a critical task, time series forecasting leverages historical sequential information to predict future trends and has been widely applied in fields such as finance, healthcare, economics, climate science, energy management, traffic control, and supply chain optimization [1]. Over recent decades, researchers have systematically investigated forecasting methodologies employing both traditional statistical techniques and machine learning approaches. Traditional statistical models typically rely on historical data characteristics such as mean, variance, and autocorrelation, while incorporating considerations of non-stationarity, linearity assumptions, and specific probability distributions. In contrast, machine learning models autonomously learn underlying patterns from data and can normally achieve better results. In the past few years, the advent of deep learning has revolutionized this domain, with increasingly sophisticated neural network architectures being applied to time series forecasting. Unlike conventional methods that necessitate domain expertise or manual feature engineering, deep learning frameworks demonstrate exceptional capability in automatically extracting intricate temporal features and dependencies from time series based on a large number of learnable parameters. This inherent capacity enables them to effectively capture long-range temporal correlations and nonlinear relationships, consequently achieving substantial improvements in prediction accuracy.

In deep learning-based time series forecasting, prior neural architectures have already focused on local or global features. These methods preserve dimensional information within the latent feature space through specialized network designs, including recurrent neural networks (RNNs) [2], convolutional neural networks (CNNs) [3], or graph neural networks (GNNs) [4]. Currently, emerging research demonstrates competitive forecasting performance using simplified linear network architectures, challenging the necessity of complex nonlinear modeling for certain time series domains [5,6].

The Transformer architecture [7] originally demonstrated exceptional performance in computer vision [8] and natural language processing (NLP) [9] tasks, has recently gained significant traction in time series analysis. Empirical evidence demonstrates its superiority over both conventional statistical methods and RNN-based architectures across various forecasting tasks [10]. Existing Transformer-based forecasting models primarily concentrate on three technical aspects: (1) sparse variable modeling [11,12], (2) self-attention mechanism correlation [13,14], and (3) cross-dimensional dependency [15]. Nevertheless, these approaches exhibit limitations in capturing lead or lag relationships between multivariate time series components, particularly when confronted with pronounced waveform variations in long-term forecasting scenarios.

To address the challenges posed by complex and highly variable features in multivariate time series, this paper proposes a scale-adaptive attention mechanism leveraging a mixture of experts framework, complemented by the novel MixNet architecture. The proposed framework facilitates flexible feature extraction across multivariate time series data. Additionally, we introduce a specialized multivariate time series embedding approach designed to capture intricate inter-variable dependencies. By integrating position-specific encoding tailored for multivariate contexts, the method enables comprehensive modeling of both intra- and inter-variable relationships, thereby achieving significant improvements in prediction performance. The main contributions of this paper are shown as follow:

- To realize flexible modeling on time series with different characteristics, we propose scale-adaptive multi-head attention. It adopts several pre-defined attention kernels with different scopes and the mixture of experts structures to analyze different temporal features from multivariate time series.
- We introduce multivariate time series embedding (MTSE) to extract dependencies among multivariate time series. MTSE aggregates temporal features from different univariate time series at the first stage. After the permutation process, MTSE can share forecasting-relevant features from different time series at the second stage. Through temporal modeling and cross-variate modeling, MixNet can achieve accurate time series forecasting effectively.
- Simulation results demonstrate that the proposed MixNet achieves significantly better performance compared with six advanced time series forecasting algorithms. It realizes the lowest mean square error and mean absolute error on most cases of tests conducted on seven datasets collected from several domains.

## Related work

### Univariate time series modeling

Univariate time series forecasting models have been extensively developed to address the challenges of single-variable prediction tasks. Oreshkin et al. [16] proposed N-Beats and demonstrated that purely deep learning architectures, devoid of time-series-specific components, can surpass traditional statistical methods. Their work also contributed to the interpretability of deep learning models for time series analysis. Li et al. [11] identified two fundamental limitations of Transformer architectures in time series forecasting: locality-agnostic attention and memory bottlenecks. To mitigate these issues, they introduced LogSparse Transformer, which integrates causal convolutions into the self-attention mechanism to achieve reduced space complexity. Wu et al. [13] addressed long-term forecasting challenges by highlighting how complex temporal patterns in distant future horizons impede reliable dependency discovery. Their proposed Autoformer incorporates an Auto-Correlation mechanism for step-wise decomposition of intricate time series patterns. In subsequent work [17], they presented TimesNet with TimeBlocks, revealing the multi-periodic nature of time series through transformation of 1D sequences into 2D tensor representations. Zeng et al. [5] critically examined Transformer efficacy in time series forecasting, attributing performance limitations to the permutation-invariant nature of self-attention mechanisms that inherently disregard temporal ordering. Their proposed LTSF-Linear framework, employing simple one-layer linear models, outperforms sophisticated Transformer-based alternatives. Zhang et al. [18] proposed a multi-scale transformer pyramid networks (MTPNet). MTPNet employs a dimension-invariant embedding and a transformer pyramid structure to capture unconstrained multi-scale temporal dependencies in multivariate time series. In subsequent research [19], they introduces the Dozerformer framework, which employs a novel sparse Dozer Attention mechanism to capture locality, seasonality, and dynamic global dependencies. Currently, prevailing approaches predominantly utilize uniform parameter sets with identical receptive field for univariate modeling. However, different time series may exhibit quite different characteristics even in the same domain. Existing approaches neglect complicated characteristics of different kinds of time series, which fails to analyze temporal features within time series and constrains forecasting performance. Therefore, modeling univariate time series dynamically and flexibly is necessary.

### Multivariate time series modeling

In the domain of multivariate time series forecasting, Zhou et al. [12] addressed the computational inefficiencies of Transformer architectures for long-sequence prediction by proposing Informer. This novel framework achieves superior performance through three innovative components: ProbSparse self-attention mechanism for reduced time complexity, self-attention distilling for memory efficiency, and generative style decoder for enhanced prediction accuracy. Nie et al. [20] explored the intersection of multivariate forecasting and self-supervised representation learning, introducing PatchTST—a channel-independent patch-based Transformer architecture. By decoupling temporal patching and channel processing mechanisms, PatchTST demonstrates improved forecasting precision compared to conventional Transformer models. Liu et al. [14] re-examined Transformer structural design for time series applications, presenting iTransformer that effectively captures multivariate correlations through refined self-attention mechanisms. The model leverages layer normalization and feedforward network modules to extract series-global representations, addressing local pattern limitations in traditional approaches. Zhang et al. [15] identified a critical gap in existing Transformer-based models regarding cross-dimensional dependency modeling. Their proposed Crossformer incorporates two key innovations: Dimension-Segment-Wise (DSW) embedding for structured feature representation, and Two-Stage Attention (TSA) layers to simultaneously capture cross-time and cross-dimension relationships. Sun et al. [6] challenged the prevailing complexity-performance paradigm by demonstrating that simple feedforward neural networks (SFNNs) can achieve state-of-the-art results in long-term univariate forecasting. Their extended multivariate SFNN variant specifically targets scenarios with strong inter-series dependencies, maintaining simplicity while accommodating multi-variable interactions. Despite these advancements, current models exhibit limitations in explicitly modeling lead or lag relationships between variables, which often exhibit distinct temporal offset patterns in real-world multivariate time series. They cannot exactly capture cross-variate dependencies among multivariate time series. Therefore, achieving accurate forecasting for multivariate time series still requires further in-depth investigation.

### Methodology

**Fig 1:**
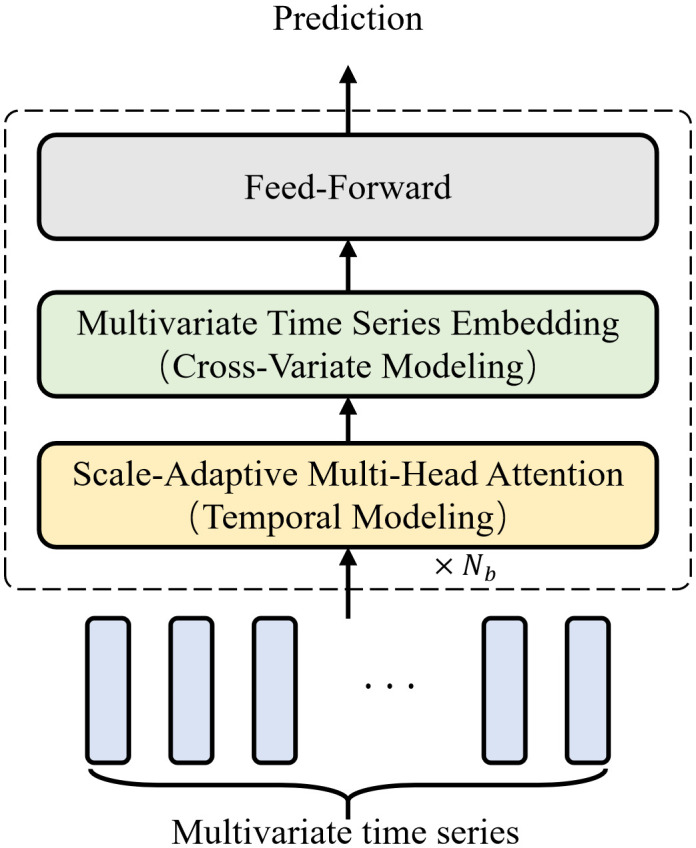
Architecture of MixNet.

In this section, we introduce MixNet, a novel architecture for multi-scale time series modeling and forecasting, as illustrated in Fig 1. *N*_*b*_ represents the number of blocks, where each block consists of a temporal modeling module, a cross-variate modeling module, and a feed-forward module. The MixNet framework introduces two core innovations to address key challenges in time series analysis: 1) a scale-adaptive multi-head attention mechanism that dynamically adjusts to time series with diverse temporal characteristics; 2) a specialized multivariate time series embedding (MTSE) method, which effectively capture complex inter-variable dependencies.

### Scale-adaptive multi-head attention

Traditional time series forecasting approaches can be broadly categorized into three methodological groups based on their feature extraction mechanisms. The first category, exemplified by N-Beats, LogSparse Transformer, and Informer, employs fully connected layers and self-attention mechanisms to extract temporal features at individual timestamps. While these methods demonstrate flexibility in capturing temporal features, they tend to overfit to forecasting-irrelevant details due to their high model capacity [20]. The second category, including Autoformer, FEDformer, and TimesNet, utilizes Fast Fourier Transform (FFT) to analyze dominant patterns in the frequency domain. However, these frequency-domain approaches face challenges in effectively capturing local temporal features and modeling non-linear dependencies across different periods [21]. The third category, represented by PatchTST and Crossformer, adopts patching strategies to achieve local feature representation while employing attention mechanisms for global feature aggregation. Nevertheless, the fixed patch size configuration limits their local perception capability and adaptability to diverse time series characteristics across various application domains.

Current attention-based time series forecasting methodologies, including PatchTST and Crossformer, utilize multi-head attention mechanisms to capture diverse temporal features. Nevertheless, real-world time series data typically exhibits inherent noise fluctuations and complex waveform patterns, which makes traditional multi-head attention cannot effectively extract temporal features, easily overfit noise, and leads to limited time series forecasting performance.

**Fig 2:**
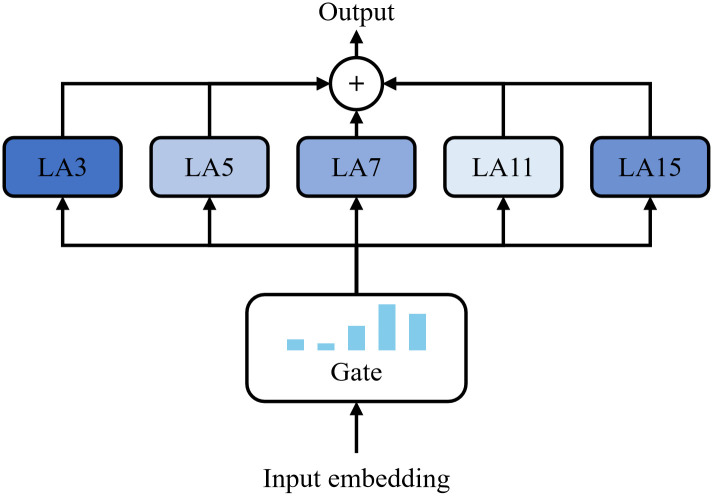
Scale-adaptive multi-head attention.

To address the limitations of existing algorithms, we introduce a scale-adaptive multi-head attention mechanism, as illustrated in Fig 2. This architecture employs multiple local attention (i.e., LA with different kernel size in Fig 2) modules with varying receptive fields to capture complex temporal features across diverse time series data. A mixture of experts framework is incorporated to dynamically assess the significance of each attention module, thereby enabling adaptive feature selection that is most relevant to the forecasting task. The output of the scale-adaptive multi-head attention for *v*-th time series, ***Y***_***v***_ is defined as


$$\begin{array}{c} Y_{v}=\sum_{i=0}^{N_{a}}w_{i,v}H_{i,v}, \end{array}$$ (1)

where $H_{i,v}$ is the temporal features extracted by the *i*-th attention module from the *v*-th time series. $w_{i,v}$ is the related weights and represents the importance the *i*-th attention module to the target time series. The weight $w_{i,v}$ and the feature $H_{i,v}$ are generated by


$$\begin{array}{c} W_{v}=\text{Gate}(X_{v}), \end{array}$$ (2)

$$\begin{array}{c} H_{i,v}=\text{Attn}(W_{i}^{Q}X_{v,t},W_{i}^{K}X_{v,t:t+r_{i}},W_{i}^{V}X_{v,t:t+r_{i}}), \end{array}$$ (3)

where $W_{v}=[w_{1,v},w_{2,v},...,w_{N_{a},v}]$ and *N*_*a*_ is the number of attention modules with each layer. Gate(·) is normally a fully connected network. $\text{Attn}(Q,K,V)=\sigma (\frac{QK}{\sqrt{d}})V$ represents the vanilla attention mechanism and $X_{v,t}$ represents the feature at position *t* of the patch sequence. *d* is the number of dimension of hidden features. $\sqrt{d}$ is used to reduce the variance in the dot product.

### Multivariate time series embedding

Multivariate time series modeling presents a significant challenge in time series analysis. The dependencies among variates can exhibit complex patterns, including synchronous or inverse fluctuations, as well as lead or lag relationships. Existing approaches can be broadly categorized into three paradigms. Currently, there are three mainstream modeling methods: (1) Informer merges all multivariate time series at the embedding stage and treat them equally. This makes it hard to distinguish features from different time series. (2) PatchTST and DLinear utilize channel independence assumption, neglecting relationships among multivariate time series. (3) Crossformer and iTransformer adopt attention mechanism to directly modeling cross-variate dependencies. However, they are also not aware of some misaligned temporal features in different time series.

**Fig 3:**
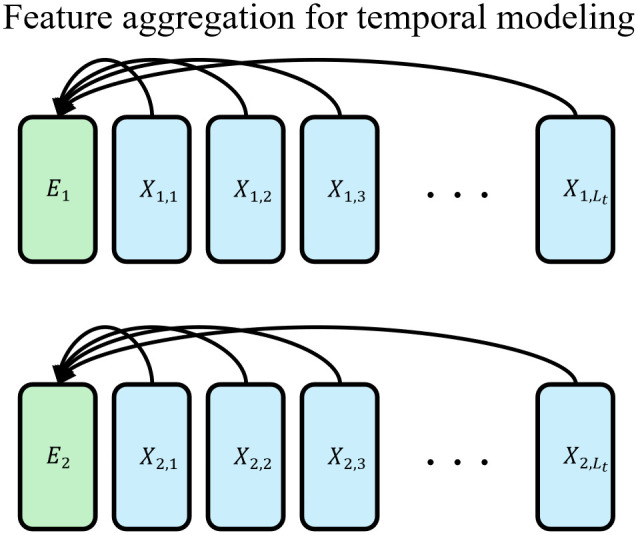
Stage 1 of multivariate time series modeling. Take time series with two variables as an example.

**Fig 4:**
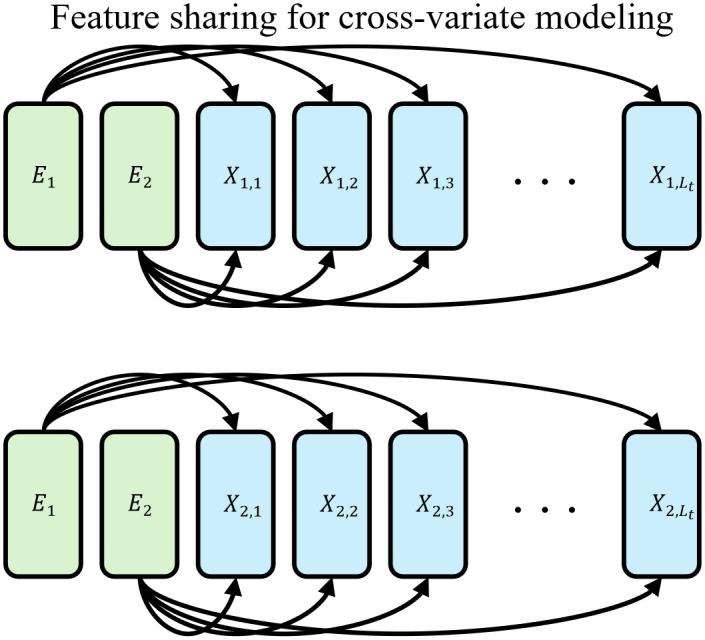
Stage 2 of multivariate time series modeling. Take time series with two variables as an example.

Inspired by the CLS token mechanism in BERT [22], we introduce MTSE to extract temporal features from individual time series, as illustrated in Figs 3 and 4. By permuting these embeddings across all time series, temporal features can be effectively shared among variates. Furthermore, the attention mechanism is employed to automatically weigh the relative importance of different variate embeddings, thereby contributing to more accurate time series forecasting. Assuming $\bar{X}$ represents the input of *v*-th time series in temporal modeling layer of a MixNet block, the first stage of MTSE is represented as


$$\begin{array}{c} {\bar{X}}_{v}=\text{MMA}(\text{concat}(E_{v},X_{v})), \end{array}$$ (4)

where MMA is the multiscale mixture attention. ***E***_*v*_ is the MTSE for the *v*-th time series. $E_{v}$ is initialized by Gaussian distribution. Let ${\hat{X}}_{v}$ denote the input of cross-variate modeling layer, the second stage of MTSE is represented as


$$\begin{array}{c} {\hat{X}}_{v}=\text{MMA}(\text{concat}([E_{1},E_{2},...,E_{N_{v}}],X_{v})). \end{array}$$ (5)

**Fig 5:**
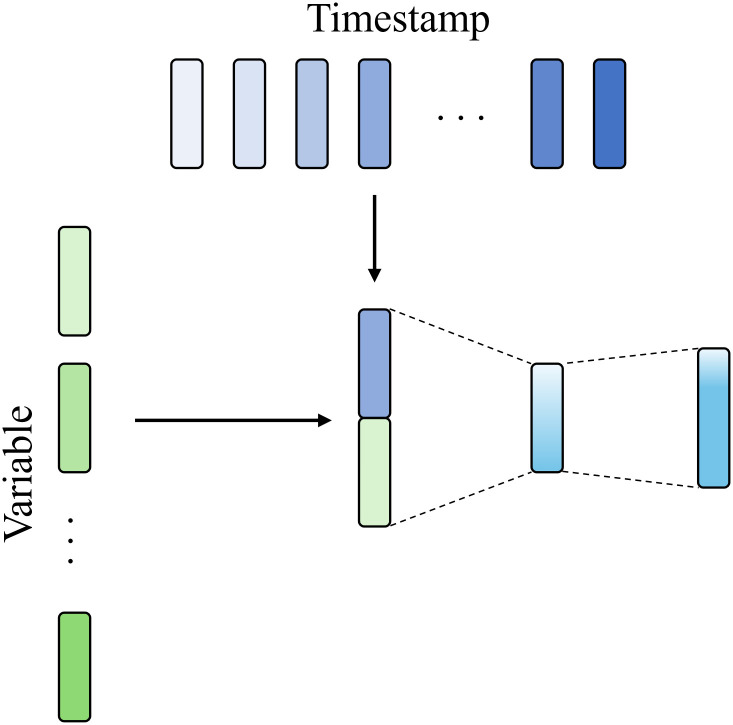
Generation of multivariate time series oriented positional encoding.

The attention mechanism is inherently permutation-invariant, which makes it difficult to discern whether the learned embeddings and features are shared across variates or are distinct, particularly when multiple time series exhibit similar features. To address this issue, we propose a multivariate time series-oriented positional encoding scheme, as depicted in Fig 5. To effectively locate features across both temporal positions and different variates, the positional encoding must possess bi-dimensional localization capability. Currently, there are two intuitive methods. One is directly defined *N*_*v*_ × *L*_*t*_ learnable vectors directly mapping all positions, where *L*_*t*_ is the length of input time series. However, this may lead to much memory consumption. The other is to defined *N*_*v*_ + *L*_*t*_ learnable vectors and locate features at different positions and different time series through combining these learnable vectors. Let ***P***_*l*_ denote the multivariate time series oriented positional encoding, the generation process is defined as


$$\begin{array}{c} P_{l}=W_{e}\text{concat}({\hat{P}}_{t},{\bar{P}}_{v}), \end{array}$$ (6)

where ***P***_*t*_ and ***P***_*v*_ represent positional encoding of temporal positions and multivariate time series separately. Both of them are vectors with learnable parameters. ***W***_*e*_ is a linear projection.

### MixNet

In the domain of time series forecasting, a variety of models with distinct architectures have been developed. The LogSparse Transformer adopts the conventional encoder-decoder architecture and generates time series in an auto-regressive manner, which, however, often suffers from error accumulation in long-sequence predictions. To mitigate this limitation, Informer introduces a generative-style decoder, substantially improving both inference efficiency and forecasting accuracy. PatchTST demonstrates much better performance with its pure decoder architecture. It utilizes a linear layer to directly forecast target time series, improving computational efficiency and reducing the risk of overfitting. Crossformer focuses on multi-scale decoding, enhancing prediction accuracy through features extracted by the encoder layers with different scales.

However, these models either have limitations in the use of features from encoders or fail to model utilize the relationships between variables at the decoder side. Motivated by these observations, we propose an innovative multi-variable iterative inference architecture that relies exclusively on the decoder. This design strategically circumvents the inherent drawbacks associated with encoder components. The architecture is designed to explicitly capture multi-scale features within each variate and to precisely extract the complex cross-variate relationships through variables with cross-variate modeling. As a result, it achieves more efficient and accurate time series forecasting.

The proposed MixNet architecture is composed of three core layers: a temporal modeling layer, a cross-variate modeling layer, and a feed-forward layer. The temporal modeling layer integrated with the scale-adaptive multi-head attention and the first stage of MTSE analyzes individual features of each time series. The cross-variate modeling layer duplicates MTSE from other time series. Based on the flexibility of the attention mechanism, the cross-variate modeling layer can analyze and utilize cross-variable dependencies for better performance. At the end of MixNet, the extracted features are projected through a fully-connected layer to generate the final forecasting results.

### Experiments

In this section, we systematically evaluate the forecasting performance of the proposed MixNet model against state-of-the-art deep learning-based forecasters through comprehensive experimental validation.

### Experiment setup

**Datasets**. Following Zhou et al. [23] and Zeng et al. [5], we conduct experiments on seven real-word datasets, including four key time series forecasting application areas: energy (ETTm1, ETTm2, ETTh1, ETTh2 and Solar), meteorology (Weather) and economics (Exchange). All of them are multivariate time series.

**Evaluation Metric**. Following previous studies [5,12,23], mean squared error (MSE) and mean absolute error (MAE) are adopted as the evaluation metrics to compare model performance.

**Baselines**. We use the following six models for multivariate time series forecasting as baselines: Dozerformer [19], iTransformer [14], PatchTST/64 [20], DLinear [5], Autoformer [13] and FEDformer [23].

**Implementation Details**. Our model is optimized using the L2 loss function and the Adam optimizer, with an initial learning rate of 0.0001 and a batch size of 32. The training employs an early stopping strategy, which halts the process if no improvement is observed within 10 epochs. All experiments are repeated for five times and implemented in PyTorch 1.8.2 [24], the mean of the metrics reported on machine with Intel(R) Xeon(R) Gold 6130 CPU @ 2.10 GHz and NVIDIA Tesla V100 GPU, from which the statistics are collected and analyzed.

### Ablation study

In this section, we conduct ablation studies to analyze the effectiveness of the MTSE. The experiments are conducted on 7 datasets. The time series from these datasets are collected from different devices with different sampling frequencies. The time series forecasting model with MTSE (Wemb) and without MTSE (W/Oemb) adopts the same model structure and hyper-parameters, like batch size, learning rate and number of dimensions are fixed in all experiments. The lengths of input time series and target time series are 96 and 336.

As shown in Table 1, Wemb achieves significantly better performance than W/Oemb in all experiments according to MSE and MAE metrics. Based on scale-adaptive multi-head attention, the temporal features of each univariate time series can be well analyzed at the first stage. The learnable MTSE in Wemb aggregates specific features of each univariate time series. After the permutation process, each time series analyzes temporal relationship together with MTSE from other time series. In addition, based on the proposed multivariate time series oriented positional encoding, features from multivariate time series can be well distinguished. Therefore, Wemb can naturally capture and utilize dependencies among different time series and achieve more accurate time series forecasting compared with W/Oemb.

**Table 1:** Multivariate time series forecasting results on 7 datasets.

Model	Wemb		W/Oemb	
Metrics	MSE	MAE	MSE	MAE
ETTh1	**0.457**	**0.448**	0.473	0.451
ETTh2	**0.418**	**0.430**	0.424	0.432
ETTm1	**0.387**	**0.400**	0.394	0.404
ETTm2	**0.300**	**0.340**	0.302	0.342
Weather	**0.262**	**0.291**	0.263	**0.291**
Exchange	**0.316**	**0.408**	0.328	0.418
Solar	**0.240**	**0.269**	0.259	0.285

Note that the best results are in bold.

### Experiment analysis

Experimental results are demonstrated in Table 2, where the lowest forecasting errors are marked in bold and the second best results are marked with underline. It is worth-noting that the proposed MixNet achieves the best and second best results in most cases. Especially, under the predict 96 setting, compared to previous state-of-the-art results, MixNet gives 7.8% MSE reduction in Weather, 2.7% MAE reduction in ETTm1. For the predict 192 setting, MixNet gives 5.2% MSE reduction and 1.9% MAE reduction in Solar. Under the predict 336 setting, MixNet gives 5.8% MSE reduction in Weather, 2.1% MAE reduction in ETTm1. For the predict 720 setting, MixNet gives 3.6% MSE reduction in Solar, 1.8% MAE reduction in Exchange. This directly shows the accuracy and robustness of the proposed MixNet in the forecasting of different kinds of time series data. The efficacy of multivariate time series forecasting models is largely determined by their proficiency in two key aspects: univariate time series modeling and cross-variate dependency mining. To address these challenges, MixNet introduces a scale-adaptive multi-head attention mechanism, which dynamically extracts abundant temporal patterns across individual time series. This design overcomes the limitation of conventional patch-based models that are confined to a fixed time period for feature extraction. Our proposed MTSE comprehensively encodes the complicated features of each time series. By concatenating these embeddings, the scale-adaptive multi-head attention mechanism can directly model relationship between different time series, and accurately achieve the extraction of cross-variate dependencies with temporal offsets.

**Table 2:** Multivariate long-term TSF results on 7 datasets.

Model		MixNet		Dozerformer		iTransformer		PatchTST		DLinear		Autoformer		FEDformer	
Metric		MSE	MAE	MSE	MAE	MSE	MAE	MSE	MAE	MSE	MAE	MSE	MAE	MSE	MAE
	96	**0.374**	0.398	0.375	**0.391**	0.386	0.405	0.414	0.419	0.386	0.400	0.449	0.459	0.376	0.419
	192	0.421	0.429	0.424	**0.420**	0.441	0.436	0.460	0.445	0.437	0.432	0.500	0.482	**0.420**	0.448
	336	**0.456**	0.448	0.464	**0.441**	0.487	0.458	0.501	0.466	0.481	0.459	0.521	0.496	0.459	0.465
ETTh1	720	**0.460**	0.467	0.470	**0.463**	0.503	0.491	0.500	0.488	0.519	0.516	0.514	0.512	0.506	0.507
	96	0.291	0.341	**0.287**	**0.339**	0.297	0.349	0.302	0.348	0.333	0.387	0.346	0.388	0.358	0.397
	192	**0.367**	**0.392**	0.369	0.393	0.380	0.400	0.388	0.400	0.477	0.476	0.456	0.452	0.429	0.439
	336	**0.420**	**0.429**	0.424	0.430	0.428	0.432	0.426	0.433	0.594	0.541	0.482	0.486	0.496	0.487
ETTh2	720	0.429	**0.444**	0.445	0.451	**0.427**	0.445	0.431	0.446	0.831	0.657	0.515	0.511	0.463	0.474
	96	**0.310**	**0.350**	0.316	0.359	0.334	0.368	0.329	0.367	0.345	0.372	0.505	0.475	0.379	0.419
	192	**0.355**	**0.379**	0.356	0.381	0.377	0.391	0.367	0.385	0.380	0.389	0.553	0.496	0.426	0.441
	336	0.387	**0.402**	**0.384**	**0.402**	0.426	0.420	0.399	0.410	0.413	0.413	0.621	0.537	0.445	0.459
ETTm1	720	**0.447**	**0.437**	0.462	0.442	0.491	0.459	0.454	0.439	0.474	0.453	0.671	0.561	0.543	0.490
	96	**0.174**	**0.257**	0.180	0.266	0.180	0.264	0.175	0.259	0.193	0.292	0.255	0.339	0.203	0.287
	192	**0.240**	**0.300**	0.251	0.311	0.250	0.309	0.241	0.302	0.284	0.362	0.281	0.340	0.269	0.328
	336	**0.300**	**0.340**	0.313	0.352	0.311	0.348	0.305	0.343	0.369	0.427	0.339	0.372	0.325	0.366
ETTm2	720	**0.397**	**0.399**	0.405	0.402	0.412	0.407	0.402	0.400	0.554	0.522	0.433	0.432	0.421	0.415
	96	**0.082**	0.202	**0.082**	**0.199**	0.086	0.206	0.088	0.205	0.088	0.218	0.197	0.323	0.148	0.278
	192	**0.173**	0.299	0.175	**0.297**	0.177	0.299	0.176	0.299	0.176	0.315	0.300	0.369	0.271	0.315
	336	0.316	0.408	**0.279**	**0.389**	0.331	0.417	0.301	0.397	0.313	0.427	0.509	0.524	0.460	0.427
Exchange	720	**0.785**	**0.665**	0.812	0.677	0.847	0.691	0.901	0.714	0.839	0.695	1.447	0.941	1.195	0.695
	96	**0.161**	**0.207**	0.162	0.213	0.174	0.214	0.177	0.218	0.196	0.255	0.266	0.336	0.217	0.296
	192	**0.206**	**0.249**	0.209	0.255	0.221	0.254	0.225	0.259	0.237	0.296	0.307	0.367	0.276	0.336
	336	**0.262**	**0.291**	**0.262**	0.295	0.278	0.296	0.278	0.297	0.283	0.335	0.359	0.395	0.339	0.380
Weather	720	0.341	0.342	**0.333**	**0.341**	0.358	0.347	0.354	0.348	0.345	0.381	0.419	0.428	0.403	0.428
	96	**0.191**	**0.235**	0.227	0.269	0.203	0.237	0.234	0.286	0.290	0.378	0.884	0.711	0.242	0.342
	192	**0.221**	**0.256**	0.266	0.296	0.233	0.261	0.267	0.310	0.320	0.398	0.834	0.692	0.285	0.380
	336	**0.240**	**0.269**	0.275	0.301	0.248	0.273	0.290	0.315	0.353	0.415	0.941	0.723	0.282	0.376
Solar	720	**0.240**	**0.271**	0.279	0.300	0.249	0.275	0.289	0.317	0.356	0.413	0.882	0.717	0.357	0.427

Note that the best and second best results are in bold and underlined, respectively.

Dozerformer achieves the second best results in most cases. Based on the local and stride components, it can easily capture various temporal features. The vary component helps it adaptively model historical time steps, enabling more accurate time series forecasting.

iTransformer achieves the third best results in most cases and is essentially an attention-enhanced Linear model. The architecture first employs linear layers to aggregate temporal features within each individual time series, and subsequently applies attention mechanisms to capture dependencies across time series. This hybrid design yields strong results in multivariate forecasting tasks. Nevertheless, it only analyze series-level relationship instead of delay or advancement relationship in temporal dimension, which may restrict its predictive capacity.

PatchTST and DLinear adopt patch-wise attention and linear layer to model temporal features within each time series separately. Both of the methods have their own advantages and disadvantages. They also adopts similar channel independence assumption for multivariate time series analysis. While this design simplifies the modeling process, it inherently disregards inter-variate dependencies, thereby limiting their forecasting capability in multivariate scenarios.

Autoformer utilizes the fast Fourier transformation based auto-correlation mechanism to analyze the time-delay similarities. FEDformer introduces frequency-enhanced attention and mixture of experts decomposition blocks to achieve temporal modeling in the frequency domain. Both of these methods can distinguish features from different time series to some extent. However, without explicit modeling dependencies among multivariate time series, they cannot achieve unsatisfactory performance.

## Conclusion

This paper presents MixNet, a novel architecture for multivariate time series forecasting. The core of our approach lies in two dedicated components, a scale-adaptive multi-head attention mechanism that leverages a mixture of experts to adaptively capture multi-scale temporal features from data with varying characteristics, and a multivariate time series embedding (MTSE) scheme that explicitly explores dependencies among variables through a structured feature aggregation and permutation process. Extensive experimental evaluations conducted on seven benchmark datasets demonstrate that the proposed MixNet outperforms several state-of-the-art baselines.

## References

[1] Benidis K, Rangapuram SS, Flunkert V, Wang Y, Maddix D, Turkmen C, et al. Deep learning for time series forecasting: tutorial and literature survey. ACM Comput Surv. 2022;55(6):1–36. doi: 10.1145/3533382

[2] Liu Y, Gong C, Yang L, Chen Y. DSTP-RNN: a dual-stage two-phase attention-based recurrent neural network for long-term and multivariate time series prediction. Expert Systems with Applications. 2020;143:113082. doi: 10.1016/j.eswa.2019.113082

[3] Lai G, Chang W-C, Yang Y, Liu H. Modeling long- and short-term temporal patterns with deep neural networks. In: The 41st International ACM SIGIR conference on research & development in information retrieval, 2018. 95–104. 10.1145/3209978.3210006

[4] Cao D, Wang Y, Duan J, Zhang C, Zhu X, Huang C. Spectral temporal graph neural network for multivariate time-series forecasting. Adv Neural Inform Process Syst. 2020;33:17766–78.

[5] Zeng A, Chen M, Zhang L, Xu Q. Are transformers effective for time series forecasting?. AAAI. 2023;37(9):11121–8. doi: 10.1609/aaai.v37i9.26317

[6] Sun FK, Wu YC, Boning DS. Simple feedforward neural networks are almost all you need for time series forecasting. 2025. doi: 10.48550/arXiv.2503.23621

[7] Vaswani A, Shazeer N, Parmar N, Uszkoreit J, Jones L, Gomez AN. Attention is all you need. Adv Neural Inform Process Syst. 2017;30.

[8] Liu Z, Lin Y, Cao Y, Hu H, Wei Y, Zhang Z, et al. Swin transformer: hierarchical vision transformer using shifted windows. In: 2021 IEEE/CVF International Conference on Computer Vision (ICCV), 2021. 9992–10002. 10.1109/iccv48922.2021.00986

[9] Zhang H, Gong Y, Shen Y, Li W, Lv J, Duan N, et al. Poolingformer: long document modeling with pooling attention. In: International conference on machine learning, 2021. 12437–46.

[10] Wen Q, Zhou T, Zhang C, Chen W, Ma Z, Yan J, et al. Transformers in time series: a survey. In: Proceedings of the Thirty-Second International joint conference on artificial intelligence, 2023. 6778–86. 10.24963/ijcai.2023/759

[11] Li S, Jin X, Xuan Y, Zhou X, Chen W, Wang YX. Enhancing the locality and breaking the memory bottleneck of transformer on time series forecasting. Adv Neural Inform Process Syst. 2019;32.

[12] Zhou H, Zhang S, Peng J, Zhang S, Li J, Xiong H, et al. Informer: beyond efficient transformer for long sequence time-series forecasting. AAAI. 2021;35(12):11106–15. doi: 10.1609/aaai.v35i12.17325

[13] Wu H, Xu J, Wang J, Long M. Autoformer: decomposition transformers with auto-correlation for long-term series forecasting. Adv Neural Inform Process Syst. 2021;34:22419–30.

[14] Liu Y, Hu T, Zhang H, Wu H, Wang S, Ma L, et al. iTransformer: inverted transformers are effective for time series forecasting. 2023. doi: 10.48550/arXiv.2310.06625

[15] Zhang Y, Yan J. Crossformer: transformer utilizing cross-dimension dependency for multivariate time series forecasting. In: International conference on learning representations. 2023.

[16] Oreshkin BN, Carpov D, Chapados N, Bengio Y. N-BEATS: Neural basis expansion analysis for interpretable time series forecasting. 2019. doi: 10.48550/arXiv.1905.10437

[17] Wu H, Hu T, Liu Y, Zhou H, Wang J, Long M. Timesnet: Temporal 2d-variation modeling for general time series analysis. 2022. 10.48550/arXiv.2210.02186

[18] Zhang Y, Wu R, Dascalu SM, Harris FC. Multi-scale transformer pyramid networks for multivariate time series forecasting. IEEE Access. 2024;12:14731–41. doi: 10.1109/access.2024.3357693

[19] Zhang Y, Wu R, Dascalu SM, Harris Jr FC. Sparse transformer with local and seasonal adaptation for multivariate time series forecasting. Scient Rep. 2024;14(1):15909. doi: 10.1038/s41598-024-66886-1 PMC11237001 38987385

[20] Nie Y, Nguyen NH, Sinthong P, Kalagnanam J. A time series is worth 64 words: Long-term forecasting with transformers. In: International conference on learning representations. 2023.

[21] Wang Y, Qiu Y, Chen P, Shu Y, Rao Z, Pan L. LightGTS: a lightweight general time series forecasting model. In: International Conference on Machine Learning, 2025. 64109–26.

[22] Zhou C, Li Q, Li C, Yu J, Liu Y, Wang G, et al. A comprehensive survey on pretrained foundation models: a history from BERT to ChatGPT. Int J Mach Learn & Cyber. 2024;16(12):9851–915. doi: 10.1007/s13042-024-02443-6

[23] Zhou T, Ma Z, Wen Q, Wang X, Sun L, Jin R. Fedformer: frequency enhanced decomposed transformer for long-term series forecasting. In: International Conference on Machine Learning, 2022. 27268–86.

[24] Paszke A, Gross S, Massa F, Lerer A, Bradbury J, Chanan G, et al. Pytorch: an imperative style, high-performance deep learning library. Adv Neural Inform Process Syst. 2019;32.

